# Irreversibility and Energy Transfer at Non-MHD Scales in a Magnetospheric Current Disruption Event

**DOI:** 10.3390/e27030260

**Published:** 2025-03-01

**Authors:** Giuseppe Consolini, Paola De Michelis

**Affiliations:** 1 INAF—Istituto di Astrofisica e Planetologia Spaziali, Via del Fosso del Cavaliere 100, 00133 Roma, Italy; 2 Istituto Nazionale di Geofisica e Vulcanologia, Via di Vigna Murata 605, 00143 Roma, Italy; paola.demichelis@ingv.it

**Keywords:** space plasma turbulence, irreversibility, energy transfer, magnetospheric current disruption

## Abstract

Irreversibility and the processes occurring at ion and sub-ion scales are key challenges in understanding energy dissipation in non-collisional space plasmas. Recent advances have significantly improved the characterization of irreversibility and energy transfer across scales in turbulent fluid-like media, using high-order correlation functions and testing the validity of certain fluctuation relations. In this study, we explore irreversibility at non-MHD scales during a magnetospheric current disruption event. Our approach involves analyzing the asymmetric correlation function, assessing the validity of a fluctuation relation, and investigating delayed coupling between different scales to reveal evidence of a cascading mechanism. The results clearly demonstrate the irreversible nature of fluctuations at ion and sub-ion scales. Additionally, we provide potential evidence for an energy cascading mechanism occurring over short time delays.

## 1. Introduction

Space plasmas are typically in an out-of-equilibrium state and are characterized by extremely low densities—on the order of just a few particles per cubic centimeter—making them effectively non-collisional. This non-collisional nature presents a fascinating challenge in understanding how such media relax toward equilibrium, i.e., how they dissipate energy. Another significant property of space plasmas is their turbulent nature [1,2]. For example, the solar wind, a turbulent plasma flow with a high Reynolds number, flows through the interplanetary medium with very low density. Despite its widespread observation, the mechanisms underlying its irreversible behavior and microscopic relaxation remain poorly understood. Unlike ordinary fluid turbulence, where viscosity dissipates energy below the inertial range and ensures irreversibility, space plasmas exhibit a different behavior. Below the inertial range, a new power-law domain emerges at ion and sub-ion scales, raising questions about whether dissipation occurs at these scales. Magnetic field fluctuations at ion/sub-ion scales follow a ∼$k^{-8/3}$ (∼$f^{-8/3}$) spectrum, which suggests global scale invariance. This characteristic appears to contrast with the strong turbulent processes typically associated with dissipation [3]. Similar features have been observed during relaxation phenomena in space plasmas, such as magnetospheric current disruption events [4] and magnetic reconnection [5,6]. Furthermore, in the case of turbulent magnetic reconnection, the importance of filamentary current sheets in determining the observed spectral features has been widely investigated from both observational and theoretical perspectives [7,8,9].

Over the past decade, there has been renewed interest in exploring irreversibility in nonequilibrium systems. Many systems subjected to external constraints exist in nonequilibrium states, exhibiting currents that provide clear evidence of broken detailed balance [10,11]. Such systems are characterized by a positive entropy production rate, which is a hallmark of time-reversal symmetry breaking.

Evaluating the entropy production rate is, however, a highly complex task, especially for real-world systems where entropy-related quantities are not easily measurable (see Ref. [12] and the references therein for a detailed discussion). In this context, an alternative approach is to study specific time-asymmetric correlation functions, which can detect time-reversal symmetry breaking. This concept was first introduced in a seminal paper by Y. Pomeau in 1982 [13]. More recently, Josserand et al. [14] and Cocciaglia et al. [15] applied these methods to turbulent systems, demonstrating how asymmetric time-correlation functions can effectively probe the irreversible dynamics and energy cascades in such systems. Another prominent method for investigating the irreversibility of dynamical systems involves fluctuation relations (or theorems). In nonequilibrium stationary states (NESS), which are defined by nonzero entropy production rates, Gallavotti and Cohen [16,17] proposed a fluctuation theorem that describes a non-trivial symmetry property of the probability distribution of phase space contraction over long timescales [18,19]. This approach has been widely applied across various physical systems to test for the presence of NESS and irreversibility [20,21,22,23].

Recently, stochastic thermodynamics has provided additional tools for characterizing entropy production in nonequilibrium processes [24,25,26]. These methods have been successfully applied to space plasma dynamics, including solar, interplanetary, and magnetospheric plasma systems, to reveal their irreversible and nonequilibrium behaviors [23,27,28].

The Earth’s magnetosphere is a complex, out-of-equilibrium system [27] that continuously interacts with the solar wind and ionosphere. This interaction gives rise to phenomena such as magnetospheric substorms and magnetic storms. These events involve various processes occurring across different regions of the magnetosphere, including plasma energization, reconfiguration, and intensification of currents.

One of the most significant phenomena occurring during the initial phase of a magnetospheric substorm is the *magnetotail current disruption* (CD) [29,30]. During this phase, a diversion or disruption of the near cross-tail current takes place, forming a current wedge that connects the central plasma sheet (CPS) of the Earth’s magnetotail to the auroral ionosphere [31,32]. This current disruption leads to an increase in the magnetic field’s $B_{Z}$-component, corresponding to a dipolarization of the magnetic field and a reduction in the cross-tail current.

Previous studies of current disruption events have demonstrated that CD is a multiscale phenomenon driven by an instability mechanism that produces broadband excitations spanning both lower [33,34] and higher frequencies. Specifically, CD involves a wide range of fluctuations, with magnetic field fluctuations displaying distinct spectral features depending on the frequency range.

At frequencies higher than the ion-cyclotron frequency $f_{\Omega }$, the magnetic field fluctuations follow a power-law spectrum, $S\left(f\right)∼f^{-\beta }$, with a spectral exponent β≃7/3÷8/3 [4,35,36,37]. Conversely, at frequencies lower than $\Omega_{c}$ the spectral features resemble those of 1/f—noise or MHD/HD turbulence. An inverse energy cascade may occur at scales and timescales larger than those associated with the drift-driven electromagnetic ion cyclotron (EMIC) instability, or ordinary mode instability, which is driven by cross-field ion drift and is thought to trigger the phenomenon [38]. Yoon et al. [39] further proposed that the observed fluctuation fields may be better explained by Alfvén ion cyclotron instability, characterized by propagation angles nearly perpendicular to the local magnetic field.

An important question arising from previous studies concerns the irreversible nature of the CD event and whether a cascading mechanism contributes to the spectral features observed at non-MHD ion and sub-ion scales. Specifically, do these spectral features result from an energy transfer from the instability scale to smaller scales via a cascading process, as seen in turbulence, or do they originate from a different mechanism?

To address these questions, methods based on the analysis of asymmetric correlation functions can be highly effective. In detail, we aim to characterize the nonequilibrium properties of CD fluctuations at sub-ion scales. In this study, we focus on how the system’s variables evolve over time and whether their correlations indicate a broken temporal symmetry, which would signal the presence of irreversible processes. By analyzing these temporal correlations, we can gain valuable insights into the underlying dynamics of out-of-equilibrium systems. More in detail, we analyze the asymmetry of the correlation functions of time increments of the magnetic field across different temporal scales, focusing on frequencies higher than the ion-cyclotron scale. It is important to remark that—although studies based on actual space measurements generally assume Taylor’s hypothesis to link temporal and spatial scales—in some situations, the validity of the correspondence between the two representations may be questionable. This is why we focus our analysis on temporal scales. Our findings reveal a clear breaking of time-reversal symmetry, which we discuss within the context of the irreversible nature of the observed phenomenon. Additionally, we investigate asymmetries in energy transfer across scales, seeking evidence of a cascading mechanism. This approach follows the methodology employed by Josserand et al. [14].

This paper is organized as follows: Section 2 presents the theoretical framework and the dataset employed in this study, detailing the methods used to analyze magnetic field fluctuations at ion and sub-ion scales during a CD event. Section 3 discusses the results, focusing on the asymmetric time-correlation functions, fluctuation theorem analyses, and potential evidence of cascading mechanisms. Finally, Section 4 provides a comprehensive discussion and concludes with insights into the irreversible dynamics and energy transfer processes, highlighting areas for future research.

## 2. Methods and Data

### 2.1. Methods: Theoretical Background

The hallmark of irreversibility in out-of-equilibrium systems is the violation of temporal symmetry, which reflects the system’s inability to spontaneously return to its initial state once disturbed. This violation is mathematically represented by the absence of detailed balance, leading to a positive entropy production rate $\sigma_{X}$, i.e.,

(1) $$\sigma_{X}=\sum_{k}J_{k}X_{k}\geq 0$$

where $J_{k}$ and $X_{k}$ are the thermodynamic fluxes and forces, respectively [10]. This concept is central to our study, as we aim to identify and quantify the irreversible dynamics of complex systems by examining temporal asymmetries. The violation of temporal symmetry, leading to a positive entropy production rate, is a fundamental characteristic of both stationary and non-stationary nonequilibrium systems. As such, a nonzero entropy production rate serves as evidence of irreversibility. However, direct measurements of the entropy production rate $\sigma_{X}$ are typically not feasible in most real-world situations. As a result, an alternative approach to testing for the occurrence of irreversibility is to investigate whether temporal symmetry is broken. This can provide valuable insights into the system’s irreversible dynamics without the need for direct entropy measurements.

In the past, Pomeau [13] introduced a method based on asymmetric correlation functions to test for temporal symmetry breaking (see also Ref. [15]).

The basic idea is rooted in the violation of the *detailed balance relation* [40,41]. Specifically, for a system in equilibrium, given two different observable state functions, *f* and *g*, the following relationship should hold due to reversibility:

(2) 〈f(t)g(0)〉=〈f(0)g(t)〉

Thus, if this equation is not true, i.e., if,

(3) 〈f(t)g(0)〉≠〈f(0)g(t)〉

then we are in an out-of-equilibrium situation and temporal symmetry is expected to be broken. Moving from Equation (3), we could use the following difference

(4) Δ(t)=〈f(t)g(0)〉−〈f(0)g(t)〉

as a measure of the distance from equilibrium and an evidence for the temporal symmetry breaking.

Following this very simple reasoning and the formalism introduced by Pomeau [13] (see also Jossrand et al. [14]), Cocciaglia et al. [15] considered the following asymmetric time-correlation function to detect the temporal symmetry breaking,

(5) $$\Psi_{x}\left(\tau \right)=\langle x^{2}\left(t\right)x\left(t+\tau \right)\rangle -\langle x\left(t\right)x^{2}\left(t+\tau \right)\rangle$$

where x(t) is a signal associated with a system observable. They applied this method to test irreversibility in fluid shell models by assuming as observable $x\left(t\right)=e_{n}\left(t\right)\equiv |u_{n}\left(t\right){|}^{2}/2$. While in the case of inviscid shell models the *detailed balance* is locally satisfied in the Fourier space,

(6) $$\Psi_{e_{n}}\left(\tau \right)=0,$$

for the forced and viscid case there is a clear violation of the *detailed balance* [15] being,

(7) $$\Psi_{e_{n}}\left(\tau \right)\neq 0,$$

The dynamics observed for the viscous case align with the concept that energy decreases over short timescales, then increases over longer times. As highlighted by Cocciaglia et al. [15], this behavior is consistent with the occurrence of *flight-crash events* [22], where *fluid elements decelerate more quickly than they accelerate*, a phenomenon familiar to anyone who has driven in heavy traffic. The trend observed in the asymmetric time-correlation function, $\Psi_{e_{n}}\left(\tau \right)$, further supports the irreversible nature of energy transfer within the cascade mechanism.

To investigate the irreversible nature of a system’s dynamics, another important quantity to examine is the statistics of energy increments, defined as W(τ)=E(t+τ)−E(t). For stationary, homogeneous flows, the first moment of this quantity is expected to vanish, i.e., 〈W(τ)〉=0, while the third moment is the first nonzero odd moment, meaning that $-\langle W^{3}\left(\tau \right)\rangle \neq 0$. However, an alternative and potentially more effective method for characterizing the irreversibility of the observed phenomenon is through the probability distribution function (PDF) of W(τ), which encapsulates all the relevant information about the energy fluctuations. To gain a deeper understanding of irreversibility, one could apply an appropriate fluctuation theorem [16,17,19] to analyze the skewness of the PDF $p\left[W(\tau )\right]$. In systems with a nonzero entropy production rate, which are out of equilibrium, fluctuation theorems assert that the probabilities of energy gain and energy loss are related by the following equation:

(8) $$\ln\left[\frac{p(-W)}{p(W)}\right]\propto W$$

which, at a first glance, is related to the shape of the tails of PDFs.

Another important aspect to explore in the context of turbulent fluctuations is the presence of a cascading process [14]. This can be examined by analyzing the following test function:

(9) $$H\left(k_{1},k_{2};\tau \right)=\langle E\left(k_{1},t_{1}\right)E\left(k_{2},t_{2}\right)\rangle -\langle E\left(k_{1},t_{2}\right)E\left(k_{2},t_{1}\right)\rangle$$

where E(k,t) is the energy at a wavenumber *k* at time *t*, τ=t2−t1, and $k_{2}\gg k_{1}$.

This quantity is expected to vanish both at τ=0 and as τ→∞, when the signals become completely decorrelated. Additionally, if H>0, this implies that $k_{2}$ is correlated with $k_{1}$ with a time delay of τ>0, meaning that energy transfers in the direction from $k_{1}$ to $k_{2}$. Conversely, for H<0, the correlation occurs in the opposite direction, with energy transferring from $k_{2}$ to $k_{1}$. By using the previously defined asymmetric correlation function H and considering three or more well-separated wavenumbers such that $k_{3}\gg k_{2}\gg k_{1}$, it becomes possible to investigate whether energy takes more time to cascade from $k_{1}$ to $k_{2}$ than from $k_{1}$ to $k_{2}$ [14]. This analysis provides valuable insights into the dynamics of energy transfer across different scales, offering potential evidence for the occurrence of a cascading mechanism. Specifically, it helps identify whether energy transfer between scales follows a hierarchical structure, which is characteristic of a turbulence-driven cascade.

### 2.2. Data

The theoretical framework outlined above was applied to analyze the statistics of magnetic field increments at ion/sub-ion scales during a specific CD event that occurred on 14 August 2013. This event was observed by the P5 (A) satellite of the THEMIS mission, which was positioned at a distance of approximately nine Earth radii (R_*E*_) in the magnetotail, within the neutral sheet, and in the pre-midnight sector. The analysis of this event builds upon previous work by Lui et al. [42], who studied the same event in the context of the dynamics of the magnetospheric current disruption. The plasma velocity is generally very small before and after CD, i.e., the plasma can be considered to be practically stagnant. However, during CD, the plasma is accelerated in a bursty manner, with a typical ion velocity on the order of ∼200÷300 km/s [43], which is close to the Alfvén velocity $c_{A}≃400$ km/s, assuming a plasma density of the order of 1 cm^−3^. Furthermore, during the CD event, the breakdown of the frozen-in condition has been observed [43].

**Figure 1:**
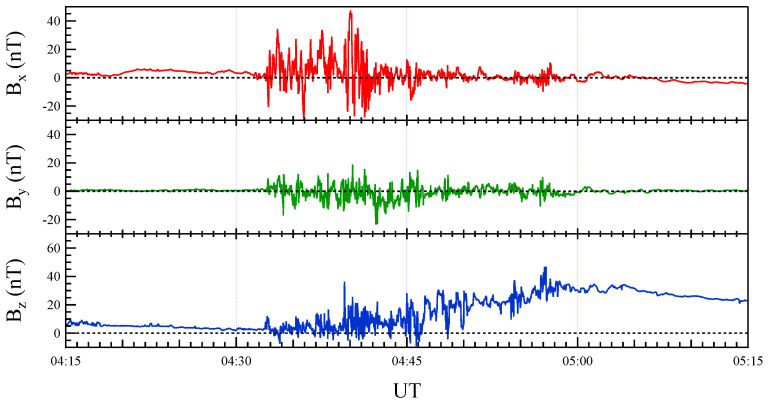
Time series of the magnetic field components ($B_{x}$, $B_{y}$, $B_{z}$) measured by the THEMIS P5 satellite during the CD event on 14 August 2013.

Figure 1 displays the three components of the magnetic field (Bx, By, Bz) measured by the THEMIS P5 satellite during the CD event on 14 August 2013. During the event, the Bz component exhibits a marked increase, indicating the onset of a dipolarization process associated with the disruption of the cross-tail current, a hallmark of CD events. Before the event, the magnetic field was nearly zero, reflecting the satellite’s position within the neutral sheet, where the field is inherently weak. After the CD, the magnetic field predominantly aligns along the Z-axis, indicating a transition to a more dipolar geomagnetic configuration in the magnetotail, signifying substantial reorganization of the field structure.

**Figure 2:**
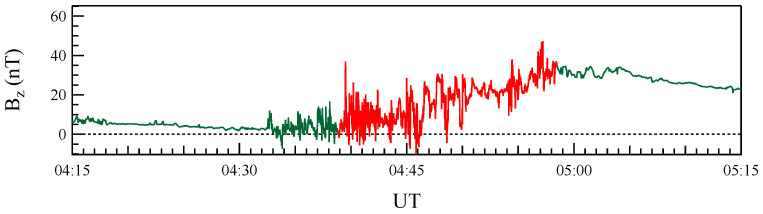
High-cadence measurements of the $B_{z}$ component of the magnetic field during the CD event, recorded by the THEMIS P5 satellite. The red trace marks the sub-interval selected for detailed analysis.

To analyze short-timescale fluctuations, we use burst-mode measurements from the fluxgate magnetometer (FGM), offering a temporal resolution of approximately 7.8 ms. These high-cadence data, central to this study, correspond to a specific sub-interval of the CD event, as shown in Figure 2. This dataset enables a detailed investigation of rapid magnetic field fluctuations during the event.

To focus on the properties of magnetic field fluctuations, long-term trends were removed from the magnetic field components using the empirical mode decomposition (EMD) technique [44]. This method ensures that each component of the magnetic field has a zero-mean value, effectively isolating the short-term fluctuations from any underlying trends. This preprocessing step is crucial for accurately analyzing the fluctuations at the ion/sub-ion scales, as it eliminates any low-frequency components that might obscure the more rapid, short timescale dynamics. Henceforward, all analyses will be performed using the detrended magnetic field time series.

**Figure 3:**
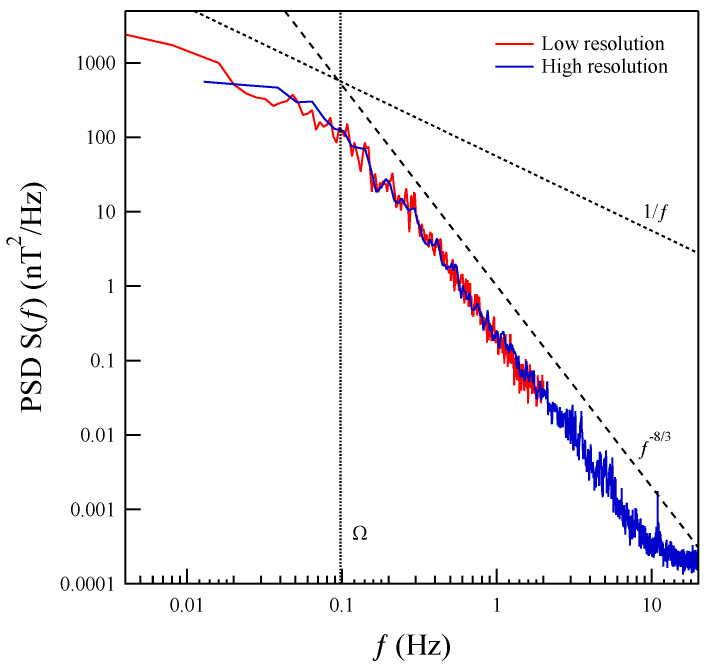
Comparison of the trace of the power spectral density (PSD), S(f), obtained from low-resolution and high-resolution (burst-mode) measurements during the CD event. The dotted and dashed lines represent power-law behaviors characterized by a 1/f and $f^{-8/3}$ behavior, respectively.

In Figure 3, we present a comparison of the trace of the power spectral density (PSD), defined as $S\left(f\right)=\sum_{i}PSD_{i}\left(f\right)$, where $PSD_{i}\left(f\right)$ represents the spectral density of the *i*-th component of the magnetic field, between the slow and burst mode measurements. This comparison highlights the differences in the spectral characteristics captured by the two measurement modes, providing insight into the behavior of the magnetic field fluctuations across different timescales.

The trace of the power spectral density (PSD), S(f), of the high-cadence data reveals a distinct power-law region, $S\left(f\right)∼f^{-\alpha }$, below the ion-cyclotron frequency ($f_{\Omega }∼0.1$ Hz). This region is characterized by a spectral exponent α≃−8/3 and extends up to approximately 8 Hz. The observed behavior is strikingly similar to the power spectrum observed in the solar wind [3]. The possible origin of the observed similarity with kinetic domain spectrum in solar wind could be an effect of large-scale random sweeping as described in Servidio et al. [45]. However, it is important to emphasize that, in the present case, we are dealing with real-time fluctuations. This distinction is crucial because, unlike in cases where Taylor’s hypothesis applies, we cannot assume the usual assumptions of spatial homogeneity or isotropy to relate the temporal and spatial characteristics of the fluctuations. This issue is critical because, when analyzing temporal fluctuations directly, we can investigate the occurrence of irreversibility, a property that has been explored in the context of shell-model turbulence [15]. This opens up the possibility of studying the irreversible nature of the system from a different perspective compared to the traditional spatial turbulence models.

## 3. Results

Following the approach outlined by Cocciaglia et al. [15] for investigating temporal symmetry breaking, we concentrate on computing the energy content at a fixed timescale, denoted as δt, in our analysis. To efficiently extract the energy corresponding to a specific timescale, we employ the wavelet transform, which is a highly effective tool for this purpose. The wavelet transform allows for the analysis of localized fluctuations in both time and frequency, making it particularly suited for capturing energy at different scales. In our analysis, we apply the continuous wavelet transform (CWT) to each detrended magnetic field component, $b_{i}$, to compute the energy content across different timescales. Specifically, the CWT of a $b_{i}\left(t\right)$ is given by the following:

(10) $$W_{\delta t}\left(t_{0}\right)=\frac{1}{\sqrt{\delta t}}\int_{-\infty }^{\infty }\psi \left(\frac{t-t_{0}}{\delta t}\right)b_{i}\left(t\right)dt,$$

where $\psi \left(\frac{t-t_{0}}{\delta t}\right)$ is a chosen wavelet function (the complex Morlet wavelet in our case [46,47]), δt is the timescale, and $t_{0}$ is the time translation. By applying this transform to the detrended magnetic field components, we can then evaluate the energy associated with each timescale, δt, and analyze how it varies over time. This process allows us to examine how energy is distributed across different timescales, which is key for studying temporal symmetry breaking and identifying potential irreversible processes in the system. In addition, by assessing the energy content at various timescales, we can explore how energy transfer occurs across different scales, which is a crucial step in understanding the dynamics of the system, particularly in the context of cascade processes in turbulence. We remark that differently from Cocciaglia et al. [15] we do not work in the Fourier *k*-space and that an association between temporal and spatial scales could be possible only knowing the corresponding dispersion relation for the excited modes (e.g., whistler waves [48]) and/or assuming that Taylor’s hypothesis may be applicable to some extent due to the high ion velocity observed during CD [43]. This issue could make more difficult the comparison of our results with previous works [15]. However, we believe that the above method remains valid regardless in terms of temporal evolution of spectral features.

In particular, we defined the total energy at the timescale δt as

(11) $$E_{i}\left(t|\delta t\right)=|W_{\delta t}\left(t\right){|}^{2}.$$

Using this quantity, we define the observable quantity, x(t) as the total energy of the fluctuation field at the timescale δt,

(12) $$x\left(t|\delta t\right)=\sum_{i=x,y,z}E_{i}\left(t|\delta t\right),$$

which represents the main focus of our study.

It is important to note that, in the case of the complex Morlet wavelet, a direct correspondence exists between the timescale δt and the frequency *f*. Specifically, by choosing as mother wavelet,

(13) $$\psi \left(t|\delta t\right)=\frac{1}{\sqrt{\delta t}}\exp\left[i\frac{2\pi t}{\delta t}-\frac{1}{2}{\left(\frac{t}{\delta t}\right)}^{2}\right],$$

this wavelet exhibits a peak at f=1/δt with a full width at half maximum (FWHM) given by Δf=f/4.

The quantity x(t) can be assumed to be equivalent to the energy of the fluctuations at the timescale δt as performed by Cocciaglia et al. [15] when analyzing the behavior of fluctuations at a fixed scale in turbulent shell models.

**Figure 4:**
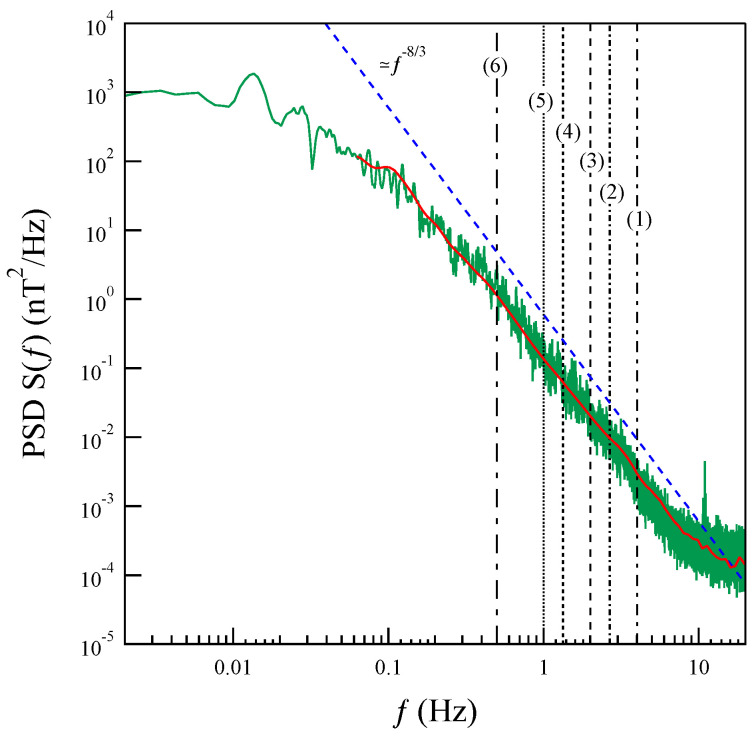
The trace of the power spectral density matrix, S(f), of the magnetic field. The vertical lines indicate the frequencies corresponding to the selected timescales τ. The red trace marks the spectra as obtained by averaging the wavelet transform in time.

Successively, we selected a series of timescales, δt, corresponding to the frequencies in the range f∈[0.5,4] Hz (refer to Table 1 and Figure 4), i.e., in the non-MHD domain.

For each selected timescale, δt, we first compute the corresponding x(t∣δt), and then calculate the following third-order correlation function:

(14) $$\Psi_{\delta t}\left(\tau \right)=\frac{\langle x^{2}\left(t\right)x\left(t+\tau \right)\rangle -\langle x\left(t\right)x^{2}\left(t+\tau \right)\rangle }{\langle x^{3}\left(t\right)\rangle }.$$

**Figure 5:**
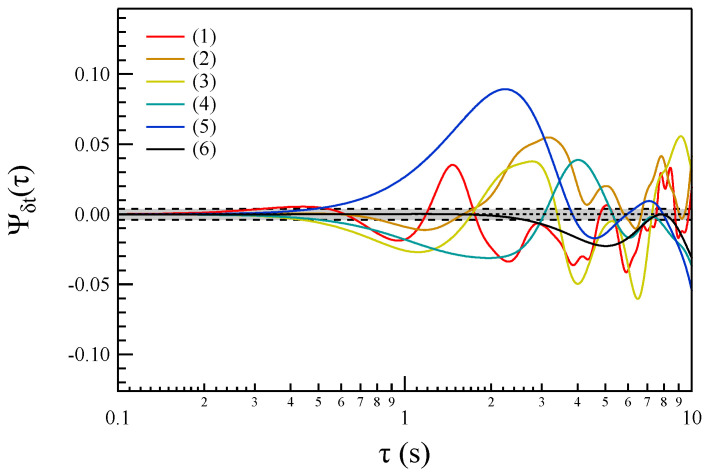
The behavior of the third-order asymmetric correlation function $\Psi_{\delta t}\left(\tau \right)$ for the selected timescales. The gray region refers to the interval for non significance.

Figure 5 illustrates the behavior of the third-order correlation function, $\Psi_{\delta t}\left(\tau \right)$, for the selected timescales listed in Table 1. The correlation function exhibits a clear time-dependent trend, which reinforces the irreversible nature of the fluctuations observed at non-MHD scales. Notably, for all the timescales under investigation, $\Psi_{\delta t}\left(\tau \right)$ shows an initial increase, followed by a rapid decrease and a subsequent rise. This pattern suggests an energy transfer originating from the characteristic timescale of the instability, which likely contributes to the broadband spectrum observed at non-MHD scales. The observed trend supports the hypothesis that the fluctuations undergo complex energy exchanges, with the energy first being pumped into the system and then released or redistributed across different timescales. This kind of behavior is characteristic of nonequilibrium processes, further confirming the irreversibility of the system dynamics. We remark that the observed trends exhibit some differences compared to the results of Cocciaglia et al. [15]. In particular, the 3rd asymmetric correlation function shows a different trend at certain temporal scales when compared with results reported in Figure 2 of Cocciaglia et al. [15]. The origin of these differences could be due to three possible different origins: (i) the phenomenon under investigation is not stationary, (ii) the spectral decomposition we use is temporal, and the applicability of Taylor’s hypothesis to associate a spatial scale may be questionable at these scales, making a direct comparison with the spatial-scale results of Cocciaglia et al. [15] not straightforward, and (iii) the statistical sample in our case is clearly more limited.

**Figure 6:**
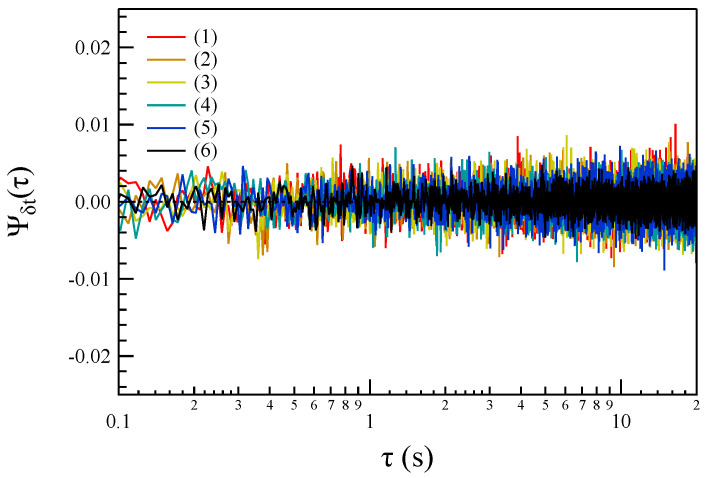
The behavior of the third-order asymmetric correlation function $\Psi_{\delta t}\left(\tau \right)$ for shuffled data at the different considered timescales.

To verify the significance of the temporal correlations observed in $\Psi_{\delta t}\left(\tau \right)$, we compute the corresponding third-order asymmetric time-correlation functions after shuffling the x(t∣δt) values. The results obtained from this procedure are presented in Figure 6.

A comparison of the results in Figure 5 and Figure 6 reveals clear evidence of temporal symmetry breaking. This observation strongly suggests the irreversible nature of the fluctuations occurring at non-MHD timescales. Additionally, the presence of temporal asymmetry in the third-order correlation function, which vanishes after data shuffling, further supports the idea that the underlying processes driving these non-MHD magnetic field fluctuations are inherently irreversible.

**Figure 7:**
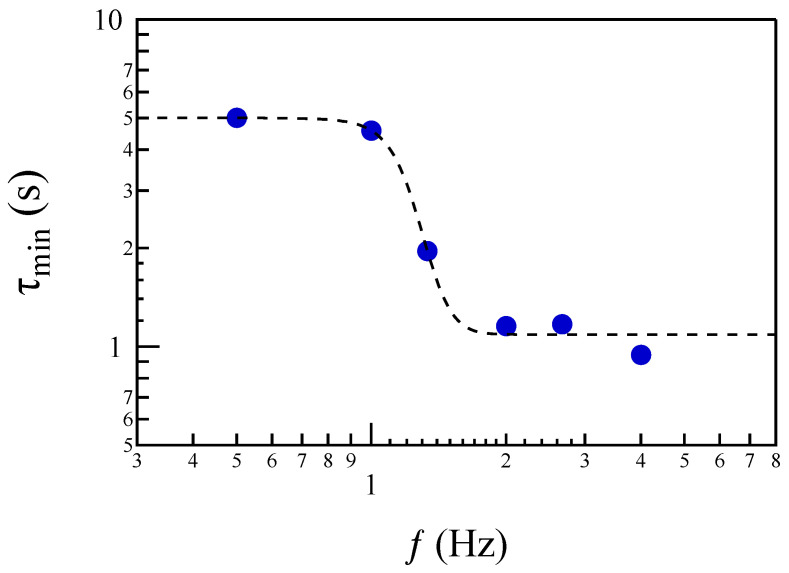
The behavior of the delay $\tau_{min}$ as a function of the corresponding frequency.

As a next step, we investigate the potential scaling behavior of the first minima in the asymmetric correlation function, $\Psi_{\delta t}\left(\tau \right)$. Figure 7 presents the trend of the delay time, $\tau_{min}$, which corresponds to the first minimum of the asymmetric correlation functions at different timescales, plotted as a function of the frequency, *f*. The trend of $\tau_{min}\left(f\right)$ is not constant; instead, it exhibits a transition-like behavior as the frequency changes. The transition occurs around a frequency of $f^{*}∼1.2$ Hz. This suggests that a dynamical transition may take place in the intermediate range between the ion and electron scales.

**Figure 8:**
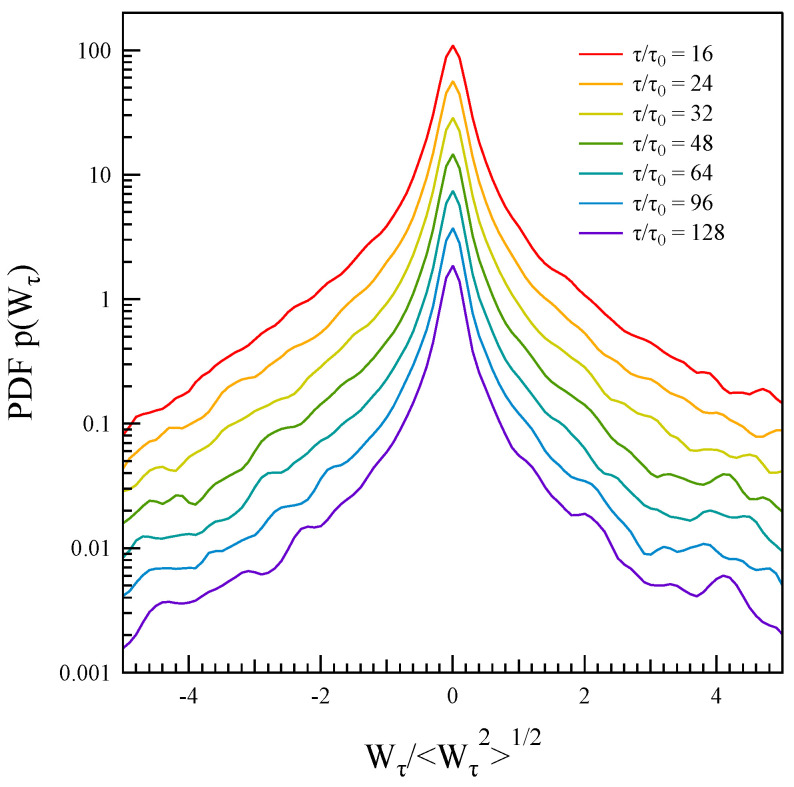
Evolution of the probability density function of the power density $p(W_{\tau })$ at different timescales τ, with the PDFs shifted for display purposes.

Following Xu et al. [22], we now examine whether the energy fluctuations at non-MHD timescales adhere to the validity of the fluctuation theorem for the magnetic field power density at timescale τ (see Equation (8)), defined as follows:

(15) $$W_{\tau }=E_{b}\left(t+\tau \right)-E_{b}\left(t\right),$$

where magnetic field power density is defined as $E_{b}\left(t\right)=\sum_{i}b_{i}^{2}/\sqrt{4\pi \rho }$ with ρ plasma density, which is assumed to be constant (so that, $E_{b}≃b^{2}$). The timescale τ is assumed to range from 0.125 to 1 s. Figure 8 illustrates the evolution of the probability density functions (PDFs) of the magnetic field power density $W_{\tau }$ at different timescales. These PDFs are leptokurtic and exhibit skewness. The positive skewness indicates that large energy increases are more probable than energy decreases. This behavior may be attributed to the explosive nature of the instability associated with the current disruption event.

By analyzing the evolution of the probability distribution function $p(W_{\tau })$ of the power density at different timescales (see Figure 8), we can attempt to estimate the prediction of the fluctuation theorem, i.e.,

(16) $$\ln\left[\frac{p(-W_{\tau })}{p(W_{\tau })}\right]≃c_{+}\left(\tau \right)\frac{W_{\tau }}{{\langle W_{\tau }^{2}\rangle }^{1/2}},$$

where $c_{+}\left(\tau \right)=c_{+}\tau$ and $W_{\tau }\leq 0$.

**Figure 9:**
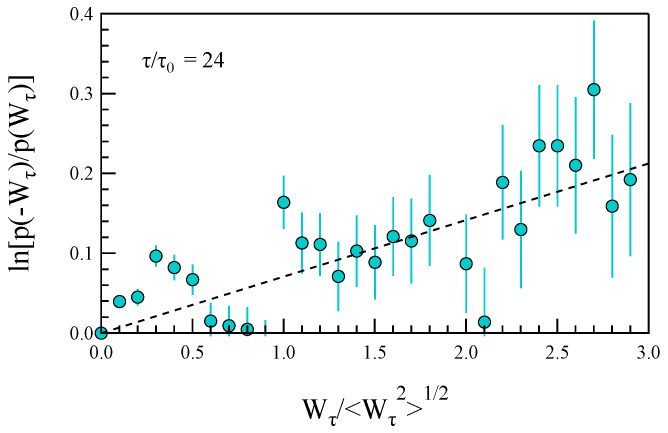
The behavior of $\ln\left[\frac{p(-W_{\tau })}{p(W_{\tau })}\right]$ for the case of $\tau /\tau_{0}=24$. The dashed line is a linear best fit.

In Figure 9, we report the behavior of $\ln\left[\frac{p(-W_{\tau })}{p(W_{\tau })}\right]$ for the case of $\tau /\tau_{0}=24$ where $\tau_{0}≃7.8$ ms is the resolution scale of magnetic field measurements. As predicted by the fluctuation theorem, a linear trend is observed on average.

**Figure 10:**
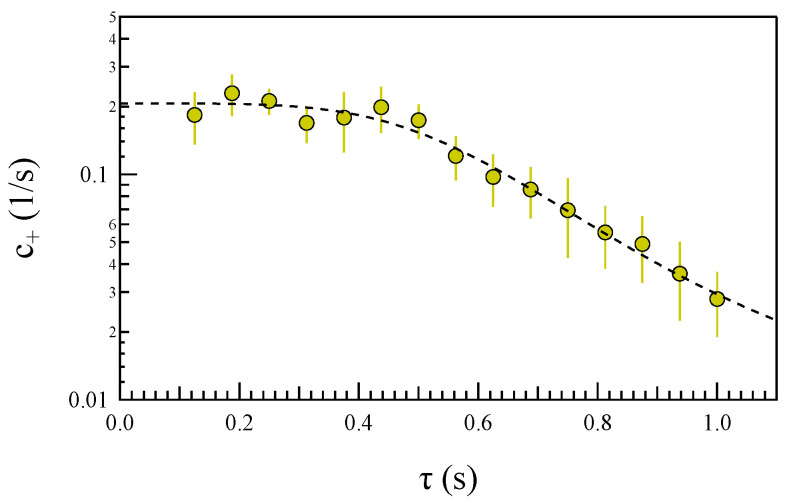
The behavior of $c_{+}$ as a function of τ. The dashed line is a guide for the eye.

For small systems in contact with thermostats, Derrida [19] suggests that the quantity $c_{+}$ should remain constant. Figure 10 shows the behavior of $c_{+}=c_{+}\left(\tau \right)/\tau$ as a function of τ. However, in contrast to the fluctuation theorem’s prediction, $c_{+}$ is not constant across the entire range of investigated scales. Instead, it decreases for τ>0.5 s. This indicates that the asymmetry in the power density distribution function diminishes as the timescale increases, approaching either the ion-cyclotron frequency $f_{\Omega }$ or the timescale of the CD instability. Consequently, the conditions outlined by the fluctuation theorem appear to hold true at scales well below the typical ion scales.

We observe that the change in the asymmetry of the distribution occurs at a timescale that aligns with the transition frequency identified in the dependence of $\tau_{min}\left(f\right)$ for the asymmetric correlation function. Specifically, assuming f∼1/2τ, we find that the scenario is consistent.

The next aspect we examine is the potential occurrence of a cascading mechanism in the energy transfer at non-MHD scales. To investigate this, we use the test function outlined in Equation (17). In our case, instead of using the wavenumber *k*, we work with the frequency *f*, and thus we compute

(17) $$H\left(f_{1},f_{2};\tau \right)=\langle E\left(f_{1},t_{1}\right)E\left(f_{2},t_{2}\right)\rangle -\langle E\left(f_{1},t_{2}\right)E\left(f_{2},t_{1}\right)\rangle$$

where $E\left(f_{i},t\right)=x\left(t|\delta t_{i}\right)$ with $f_{i}=1/\delta t_{i}$, and $t_{2}-t_{1}=\tau$. Clearly, our analysis is strictly temporal so that we cannot exclude the possibility that space-time correlation might be affected by a simply temporal-based approach. However, if we take into account that during CD plasma acceleration events are observed [43] and the plasma cannot be considered stagnant, then we are confident that the investigation of $H(f_{1},f_{2};\tau )$ could provide some information on the occurrence of cascading (energy transfer) mechanisms also in *k*-space. A multipoint analysis would be very useful in this framework.

**Figure 11:**
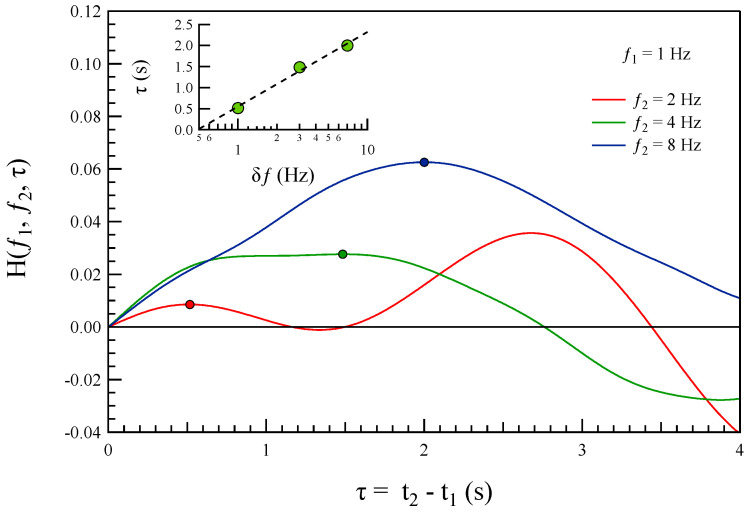
The behavior of the test function $H(f_{1},f_{2};\tau )$ for three cases studied. The dots indicate the position of the maxima. The inset shows τ(δf) where $\delta f=f_{2}-f_{1}$. Dashed line is a logarithmic fit.

In our analysis, we set $f_{1}=1$ Hz and consider $f_{2}=2$, 4, and 8 Hz. Figure 11 illustrates the test function $H(f_{1},f_{2};\tau )$ for the three cases examined. We limit our analysis to the first maxima of the test function $H(f_{1},f_{2};\tau )$. The position of the maxima, $\tau_{max}$, as a function of the frequency difference $\delta f=f_{2}-f_{1}$, provides evidence for the potential occurrence of a cascading energy transfer mechanism at non-MHD scales, at least on short timescales.

Indeed, we found that the first maxima occur in a sequence of increasing frequencies, $f_{2}<f_{3}<f_{4}$. This suggests that the initial energy transfer from $f_{1}$ to $f_{2}$ is followed by a second transfer from $f_{1}$ to $f_{3}>f_{2}$, and a third transfer from $f_{1}$ to $f_{4}>f_{3}$. Additionally, as shown in the inset of Figure 11, the timings corresponding to the first maxima at different frequencies follow a logarithmic trend as a function of the frequency difference $\Delta f=f_{i}-f_{1}$.

## 4. Discussion and Conclusions

The analysis of the asymmetric time-correlation function, $\Psi_{\delta t}\left(\tau \right)$, across various timescales (δt) within the ion and sub-ion scales (non-MHD domain, i.e., $f>\Omega_{c}$), reveals a clear violation of the detailed balance equation at these scales. This violation is a strong indicator of the irreversible nature of the fluctuations in this frequency range, suggesting that the system is far from equilibrium and dominated by nonequilibrium dynamics.

This irreversible behavior is consistent with the expected characteristics of energy transfer or dissipation processes occurring at ion/sub-ion scales. Moreover, these findings are corroborated by results obtained from shuffled data, which serve as a control test to confirm that the observed asymmetry is not due to random noise or statistical artifacts. The combined evidence points to the presence of fundamental mechanisms breaking time-reversal symmetry, further underscoring the unique and complex dynamical processes occurring in this non-MHD regime.

**Figure 12:**
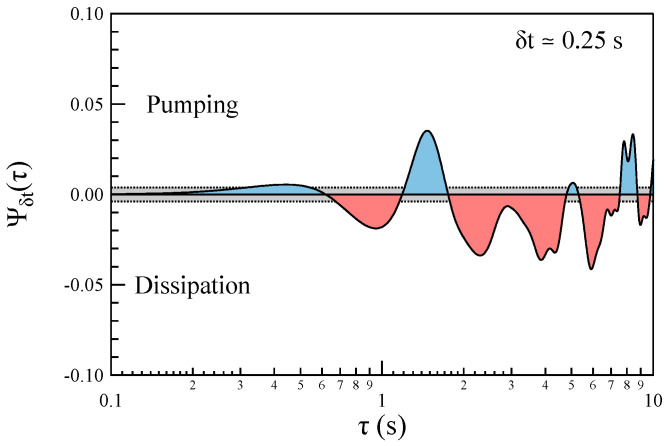
The behavior of the asymmetric correlation function for δt=0.125 s. The dashed lines delimit the non-significance range, which is marked in gray.

However, in contrast to the behavior observed in the inertial range by Cocciaglia et al. [15], the asymmetric time-correlation function $\Psi_{\delta t}\left(\tau \right)$ at certain timescales δt initially shows an increase, followed by a decrease (see Figure 5). This behavior could be a consequence of the explosive nature of the CD instability, which initially pumps energy into the system. Following this initial energy injection, the energy is then released through a damping or transfer mechanism. This is particularly evident in Figure 12, where for the smallest timescale studied (δt=0.25 s), the time-correlation function $\Psi_{\delta t}\left(\tau \right)$) is initially positive, suggesting an increase in energy at this timescale. Subsequently, it becomes negative, indicating that the energy is released. This transition from positive to negative could reflect a typical energy redistribution process in nonequilibrium systems, where energy is initially accumulated and then dissipated or transferred across scales. Such behavior highlights the dynamic and transient nature of the processes occurring during the current disruption event.

In a previous study, Consolini and Lui [49] investigated the occurrence of three-wave coupling in magnetic field fluctuations and found evidence of short-lived nonlinear interactions as current disruption progresses. This intermittent coupling aligns well with the sporadic nature of energy pumping observed through the asymmetric correlation function. Specifically, the energy transfer from the instability timescale to the non-MHD scales appears to occur intermittently, rather than in a continuous manner. This intermittent behavior suggests that the process of energy transfer is not steady but fluctuates over time, possibly driven by the dynamics and evolving nature of the current disruption event. The periodicity and short-lived nature of the coupling imply that the system undergoes transient states, where energy is rapidly transferred across different scales, before being damped or redistributed. These findings reinforce the idea that the energy dynamics during the CD event are complex and governed by nonlinear processes that are not only spatially multiscale but also temporally intermittent.

An investigation into the validity of the fluctuation theorem (FT) for the probability distribution function $p(W_{\tau })$ of the power density revealed that the FT requirements for NESS are only partially met. Specifically, while the scaling of the relative probabilities of positive and negative $W_{\tau }$ is satisfied on average, the coefficient $c_{+}\left(\tau \right)$ does not exhibit a linear trend with respect to τ. Notably, linearity seems to hold only for timescales τ<0.5 s. This finding suggests the possible occurrence of a dynamical transition, as indicated by the behavior of the first minima in the asymmetric correlation functions.

Assuming that the frequency $f^{*}≃1.2$ Hz at which we observe the dynamical transition corresponds to the ion inertial length via the Alfvén velocity $c_{A}$, i.e., $c_{A}=d_{i}f^{*}$, we can estimate a magnetic field intensity B≃10 nT. This value is of the same order as the magnetic field intensity observed during the initial phase of the current disruption (CD). Based on this, we can hypothesize that the dynamical transition occurs at a scale close to the ion inertial length $d_{i}$. This implies that the dissipation and irreversibility associated with the CD event are primarily occurring at scales smaller than $d_{i}$, i.e., at the sub-ion scales. At these smaller scales, the physical processes responsible for energy dissipation and the breakdown of detailed balance become more pronounced. Consequently, the transition to irreversible dynamics could be linked to the scale where the ion inertial length becomes relevant, marking a shift in the energy redistribution mechanisms and the onset of turbulence-like behavior. This observation further supports the idea that dissipation is predominantly occurring in the non-MHD regime, where interactions at sub-ion scales play a key role.

Furthermore, the fact that the symmetries predicted by the fluctuation theorem (FT) are only partially satisfied, a property already observed in turbulence [22], could be related to the nature of the forces acting on the plasma during the CD event. A key point to emphasize is the potential role of nonstationarity in causing the deviation from the FT predictions. Indeed, the CD event may be viewed as a transition between two nonequilibrium stationary states, where the system is evolving and not in a steady state, potentially leading to the observed discrepancies.

Another important consideration is whether a cascading mechanism is responsible for transferring energy at sub-ion scales. Although based on temporal measurements our preliminary analysis suggests that such a mechanism might be occurring. Specifically, by using the test function $H(f_{1},f_{i},\tau )$, we found evidence of time-ordering in the coupling between frequencies, with $f_{1}$ coupling to $f_{2}<f_{3}<f_{4}$. However, it is crucial to emphasize that a more thorough assessment of the potential cascading mechanism requires further analysis [14], which could also be capable of resolving spatial scales. We are confident that multipoint measurements from future space missions, such as the NASA HelioSwarm mission or the proposed ESA Plasma Observatory, can help overcome some of the limitations imposed here by purely temporal measurements in unveiling the occurrence of a clear cascading process.

For instance, when examining the test function over a longer time interval, such as τ up to 30 s, we observed moments where the coupling between different timescales appeared nearly instantaneous. This suggests a rapid energy transfer from $f_{1}$ to the other frequencies, which would contradict the existence of a cascading process, as the time-ordering would be lost. One possible explanation for this behavior is the role of instability, and potentially secondary instabilities, in the current disruption event (CD). These instabilities could be driving the observed rapid coupling between timescales, further complicating the identification of a cascading mechanism. However, given our analysis on the time domain, we cannot exclude that non-trivial space time correlations might affect the observed behavior. Thus, a confirmation of this point requires more accurate studies.

In conclusion, this study explores the potential of the asymmetric time-correlation function $\Psi_{\delta t}\left(\tau \right)$ as a tool for detecting irreversibility in space plasma processes at the ion and sub-ion scales. Our findings confirm the emergence of irreversibility in the fluctuations occurring during CD. However, we observed some key differences when compared to well-studied cases of systems in contact with thermostats, particularly in the application of fluctuation theorems (FTs). These differences suggest that nonequilibrium processes, such as turbulence which involves multiple scales, may exhibit distinct features and symmetries in their irreversible dynamics. Additionally, our results point to the possible occurrence of a cascading mechanism that transfers energy across sub-ion scales over short time intervals. While this observation provides promising evidence of such a mechanism, further investigation is required to fully validate this hypothesis. Future studies will need to explore this phenomenon in more detail, taking into account the complexities of the underlying dynamics and the potential influence of instability mechanisms, which may play a crucial role in shaping the energy transfer processes. Overall, this work highlights the value of using time-asymmetric correlation functions to probe the irreversible nature of space plasma phenomena and opens avenues for further research into the multiscale processes driving energy dissipation and transfer in space plasma systems. In particular, we believe that approaches like the ones presented in this work may be relevant in several different plasma context, such as the study of magnetic reconnection in decaying turbulence (see, e.g., [50,51]), as well as in the investigation of time-symmetry breaking phenomena in the analysis of nonlinear kinetic Vlasov–Maxwell simulations, which should formally preserve entropy [52,53].

## Figures and Tables

**Table 1:** The selected timescales (δt) and the corresponding frequencies *f*, computed as f=1/δt.

Label	δt	f=1/δt
	(s)	(Hz)
(1)	0.25	4
(2)	0.375	8/3
(3)	0.5	2
(4)	0.75	4/3
(5)	1	1
(6)	2	0.5

## Data Availability

THEMIS data used in this study are available at the NASA Space Physics Data Facility (SPDF) (https://cdaweb.gsfc.nasa.gov/index.html) accessed on 12 September 2024.

## Abbreviations

The following abbreviations are used in this manuscript:

CD	Current disruption
CPS	Central plasma sheet
FT	Fluctuation Theorem
FWHM	Full width half maximum
HD	Hydro-Dynamic
MHD	Magneto-Hydro-Dynamic
NESS	Nonequilibrium stationary state
PDF	Probability density function
PSD	Power spectral density

## References

[1] Bruno R., Carbone V. (2013). The solar wind as a turbulence laboratory. Living Rev. Sol. Phys..

[2] Zhou Y., Matthaeus W.H., Dmitruk P. (2004). Colloquium: Magnetohydrodynamic turbulence and time scales in astrophysical and space plasmas. Rev. Mod. Phys..

[3] Kiyani K.H., Chapman S.C., Khotyaintsev Y.V., Dunlop M.W., Sahraoui F. (2009). Global Scale-Invariant Dissipation in Collisionless Plasma Turbulence. Phys. Rev. Lett..

[4] Consolini G., Kretzschmar M., Lui A., Zimbardo G., Macek W. (2005). On the magnetic field fluctuations during magnetospheric tail current disruption: A statistical approach. J. Geophys. Res. Space Phys..

[5] Eastwood J., Phan T., Bale S., Tjulin A. (2009). Observations of turbulence generated by magnetic reconnection. Phys. Rev. Lett..

[6] Consolini G., Grandioso S., Yordanova E., Marcucci M., Pallocchia G. (2015). Statistical and scaling features of fluctuations in the dissipation range during a reconnection event. Astrophys. J..

[7] Servidio S., Carbone V., Primavera L., Veltri P., Stasiewicz K. (2007). Compressible turbulence in Hall magnetohydrodynamics. Planet. Space Sci..

[8] Greco A., Perri S., Servidio S., Yordanova E., Veltri P. (2016). The complex structure of magnetic field discontinuities in the turbulent solar wind. Astrophys. J. Lett..

[9] Consolini G., Alberti T., Benella S., Papini E., Pezzi O. (2023). On the fractal pattern of the current structure at ion scales in turbulent space plasmas. Chaos Solitons Fractals.

[10] De Groot S., Mazur P. (1984). Non-Equilibrium Thermodynamics.

[11] Livi R., Politi P. (2017). Nonequilibrium Statistical Physics: A Modern Perspective.

[12] Lucente D., Baldovin M., Cecconi F., Cencini M., Cocciaglia N., Puglisi A., Viale M., Vulpiani A. (2024). Conceptual and practical approaches for investigating irreversible processes. arXiv.

[13] Pomeau Y. (1982). Symétrie des fluctuations dans le renversement du temps. J. Phys..

[14] Josserand C., Le Berre M., Lehner T., Pomeau Y. (2017). Turbulence: Does energy cascade exist?. J. Stat. Phys..

[15] Cocciaglia N., Cencini M., Vulpiani A. (2024). Nonequilibrium statistical mechanics of the turbulent energy cascade: Irreversibility and response functions. Phys. Rev. E.

[16] Gallavotti G., Cohen E.G.D. (1995). Dynamical ensembles in nonequilibrium statistical mechanics. Phys. Rev. Lett..

[17] Gallavotti G., Cohen E.G.D. (1995). Dynamical ensembles in stationary states. J. Stat. Phys..

[18] Lebowitz J.L., Spohn H. (1999). A Gallavotti–Cohen-type symmetry in the large deviation functional for stochastic dynamics. J. Stat. Phys..

[19] Derrida B. (2007). Non-equilibrium steady states: Fluctuations and large deviations of the density and of the current. J. Stat. Mech. Theory Exp..

[20] Ciliberto S., Garnier N., Hernandez S., Lacpatia C., Pinton J.F., Chavarria G.R. (2004). Experimental test of the Gallavotti–Cohen fluctuation theorem in turbulent flows. Phys. A Stat. Mech. Its Appl..

[21] Shang X.D., Tong P., Xia K.Q. (2005). Test of steady-state fluctuation theorem in turbulent Rayleigh-Bénard convection. Phys. Rev. E.

[22] Xu H., Pumir A., Falkovich G., Bodenschatz E., Shats M., Xia H., Francois N., Boffetta G. (2014). Flight–crash events in turbulence. Proc. Natl. Acad. Sci. USA.

[23] Viavattene G., Consolini G., Giovannelli L., Berrilli F., Del Moro D., Giannattasio F., Penza V., Calchetti D. (2020). Testing the Steady-State Fluctuation Relation in the Solar Photospheric Convection. Entropy.

[24] Sekimoto K. (2010). Stochastic Energetics.

[25] Seifert U. (2012). Stochastic thermodynamics, fluctuation theorems and molecular machines. Rep. Prog. Phys..

[26] Peliti L., Pigolotti S. (2021). Stochastic Thermodynamics: An Introduction.

[27] Consolini G., De Michelis P., Tozzi R. (2008). On the Earth’s magnetospheric dynamics: Nonequilibrium evolution and the fluctuation theorem. J. Geophys. Res. Space Phys..

[28] Stumpo M., Benella S., Alberti T., Pezzi O., Papini E., Consolini G. (2023). Relating Intermittency and Inverse Cascade to Stochastic Entropy in Solar Wind Turbulence. Astrophys. J. Lett..

[29] Takahashi K., Zanetti L., Lopez R., McEntire R., Potemra T., Yumoto K. (1987). Disruption of the magnetotail current sheet observed by AMPTE/CCE. Geophys. Res. Lett..

[30] Lui A., Lopez R., Krimigis S., McEntire R., Zanetti L., Potemra T. (1988). A case study of magnetotail current sheet disruption and diversion. Geophys. Res. Lett..

[31] Ahn B.H., Kamide Y., Kroehl H., Candidi M., Murphree J.S. (1995). Substorm changes of the electrodynamic quantities in the polar ionosphere: CDAW 9. J. Geophys. Res. Space Phys..

[32] Lui A., Volwerk M., Dunlop M., Alexeev I., Fazakerley A., Walsh A., Lester M., Grocott A., Mouikis C., Henderson M. (2008). Near-Earth substorm features from multiple satellite observations. J. Geophys. Res. Space Phys..

[33] Ohtani S., Higuchi T., Lui A., Takahashi K. (1995). Magnetic fluctuations associated with tail current disruption: Fractal analysis. J. Geophys. Res. Space Phys..

[34] Lui A., Najmi A.H. (1997). Time-frequency decomposition of signals in a current disruption event. Geophys. Res. Lett..

[35] Kozak L., Petrenko B., Lui A., Kronberg E., Grigorenko E., Prokhorenkov A. (2018). Turbulent processes in the Earth’s magnetotail: Spectral and statistical research. Ann. Geophys..

[36] Kozak L., Petrenko B., Kronberg E., Grigorenko E., Lui E., Cheremnykh S. (2018). Spectra of Turbulence during the Dipolarization of the Magnetic Field. Kinemat. Phys. Celest. Bodies.

[37] Kozak L., Petrenko B., Lui A., Kronberg E., Daly P. (2021). Processes in the current disruption region: From turbulence to dispersion relation. J. Geophys. Res. Space Phys..

[38] Lui A., Yoon P., Mok C., Ryu C.M. (2008). Inverse cascade feature in current disruption. J. Geophys. Res. Space Phys..

[39] Yoon P., Lui A., Bonnell J. (2009). Identification of plasma instability from wavelet spectra in a current disruption event. J. Geophys. Res. Space Phys..

[40] Onsager L. (1931). Reciprocal relations in irreversible processes. I. Phys. Rev..

[41] Onsager L. (1931). Reciprocal relations in irreversible processes. II. Phys. Rev..

[42] Lui A., Mitchell D., Lanzerotti L. (2014). Comparison of energetic electron intensities outside and inside the radiation belts. J. Geophys. Res. Space Phys..

[43] Lui A. (2011). Reduction of the cross-tail current during near-Earth dipolarization with multisatellite observations. J. Geophys. Res. Space Phys..

[44] Huang N.E., Wu Z. (2008). A review on Hilbert-Huang transform: Method and its applications to geophysical studies. Rev. Geophys..

[45] Servidio S., Carbone V., Dmitruk P., Matthaeus W. (2011). Time decorrelation in isotropic magnetohydrodynamic turbulence. Europhys. Lett..

[46] Torrence C., Compo G.P. (1998). A practical guide to wavelet analysis. Bull. Am. Meteorol. Soc..

[47] Mallat S. (1999). A Wavelet Tour of Signal Processing.

[48] Zhang W., Fu H., Cao J., Wang Z., Fu W., Liu Y., Yu Y. (2024). First determination of whistler wave dispersion relation in superhot (Te > 5 keV) plasmas. Phys. Rev. Res..

[49] Consolini G., Lui A. (2000). Symmetry breaking and nonlinear wave-wave interaction in current disruption: Possible evidence for a phase transition. Geophys. Monogr.-Am. Geophys. Union.

[50] Franci L., Cerri S.S., Califano F., Landi S., Papini E., Verdini A., Matteini L., Jenko F., Hellinger P. (2017). Magnetic reconnection as a driver for a sub-ion-scale cascade in plasma turbulence. Astrophys. J. Lett..

[51] Cerri S.S., Califano F. (2017). Reconnection and small-scale fields in 2D-3V hybrid-kinetic driven turbulence simulations. New J. Phys..

[52] Ghizzo A., Del Sarto D. (2021). Momentum transfer driven by fluctuations in relativistic counter-propagating electron beams. Plasma Phys. Control. Fusion.

[53] Ghizzo A., Del Sarto D., Betar H. (2023). Collisionless heating driven by Vlasov filamentation in a counterstreaming beams configuration. Phys. Rev. Lett..

